# Comprehensive investigation of tobacco leaves during natural early senescence via multi-platform metabolomics analyses

**DOI:** 10.1038/srep37976

**Published:** 2016-11-29

**Authors:** Lili Li, Jieyu Zhao, Yanni Zhao, Xin Lu, Zhihui Zhou, Chunxia Zhao, Guowang Xu

**Affiliations:** 1 Key Laboratory of Separation Science for Analytical Chemistry, Dalian Institute of Chemical Physics, Chinese Academy of Sciences, Dalian 116023, China; 2 University of Chinese Academy of Sciences, Beijing, 100049, China

## Abstract

Senescence is the final stage of leaf growth and development. Many different physiological activities occur during this process. A comprehensive metabolomics analysis of tobacco middle leaves at 5 different developmental stages was implemented through multi-platform methods based on liquid chromatography, capillary electrophoresis and gas chromatography coupled with mass spectrometry. In total, 412 metabolites were identified, including pigments, sterols, lipids, amino acids, polyamines, sugars and secondary metabolites. Dramatic metabolic changes were observed. Firstly, membrane degradation and chlorophyll down-regulation occurred after the 50% flower bud stage. Levels of major membrane lipids decreased, including those of the glycolipids in chloroplast thylakoids and phospholipids in membrane envelopes. Clear decreases in free sterols and acylated sterol glucosides were detected along with the accumulation of sterol esters. The accumulation of alkaloids was found. The amino acid levels were significantly decreased, particularly those of N-rich amino acids (glutamine and asparagine), thus reflecting N translocation. Subsequently, the antioxidant system was activated. Sugar alcohols and polyphenols accumulated when the lower leaves turned yellow. These results comprehensively revealed the metabolic changes that occur during tobacco leaf development and senescence under natural conditions.

The leaf is an organ that conducts photosynthesis and plays a vital role in plant development. Leaf development encompasses different stages. Initially, young leaves undergo rapid expansion by absorbing nutrients and synthesizing proteins to achieve efficient photosynthesis, and then, they enter into stages of maturation and senescence^1^,^2^. Senescence is the final stage of leaf development. During leaf senescence, macromolecules, such as proteins and nucleic acids, are degraded^3^. This degradation process in leaf results in decreased photosynthesis, crop yield and plant biomass production^4^. Leaf senescence is not merely a process during which cell function deteriorates; instead, it also facilitates the mobilization of nutrients from senescing leaves to young tissues and reproducible organs. For example, the carbon, potassium, nitrogen, phosphorus and sulfur contents are reduced by more than 40% in senescing leaves of *Arabidopsis thaliana*^5^. The study of leaf senescence can improve understanding of the mechanisms of plant development and provide guidance for improving agriculture production.

Leaf senescence is a type of programmed cell death, a highly self-destructive cellular process^6^. To date, studies on leaf senescence have achieved great progress by using genetics and transcriptomics. A number of senescence-associated genes (SAGs) have been discovered in *Arabidopsis thalian*a, which participate in energy supply, macromolecular degradation and recycling, amino acid transport, and signaling and regulatory pathways^7^. Through use of the staygreen mutant, a SAG encoding a novel chloroplast protein regulating chlorophyll degradation has been identified, thus revealing the correlation between chlorophyll degradation and a light-harvesting, chlorophyll-binding protein^8^. As end products of genetic expression, metabolites also play a vital role in leaf senescence. Tocopherols are an important class of amphiphilic antioxidants in thylakoid membranes. A tocopherol deficiency restricts sugar export from the source leaves and causes premature senescence^9^. Leaf senescence can be induced or suppressed by different phytohormones. Ethylene induces senescence in leaves that have grown to a certain stage, and the effect increases with the degree of aging^10^. The production of cytokinins inhibits the breakdown of chlorophyll, maintains the rubisco and photosynthesis rates, and delays the senescence process^11^. Sugars are important regulators of plant development. Trehalose 6-phosphate levels increase 15-fold in old leaves compared with younger leaves^12^. Reactive oxygen species are produced at the onset of leaf senescence. The accumulation of free radicals increases the levels of lipid peroxidation and membrane permeability^13^. More than 60 lipases are up-regulated and catalyze the degradation of glycolipids into fatty acids^14^. Additionally, membrane function decreases as the membrane structure decomposes and further influences metabolic pathways, such as photosynthesis, photorespiration and respiration.

Metabolomics is a rapidly developing technology of the post-genomics era that focuses on global changes in biological samples. Metabolomics analyses have been conducted to determine the metabolic differences in plants under abiotic and biotic stress and have achieved great progress in exploring new physiological functions. Capillary electrophoresis (CE), gas chromatography (GC) and liquid chromatography (LC) coupled with mass spectrometry (MS) are often used in metabolomics studies^15^,^16^. CE-MS and GC-MS are suitable for the analysis of polar and highly volatile metabolites, whereas LC-MS is usually performed to analyze weakly polar metabolites. Although many studies have investigated leaf senescence, most have focused on a specific class of metabolites (e.g., sugars^12^ or polyamines^17^) under stress conditions (e.g., heat stress^18^ or external phytohormone stress^11^). To date, the comprehensive metabolomics analyses on natural leaf senescence is still rare. Tobacco is an economic crop and also an important model plant. Tobacco has been widely applied in plant biological research. For example, nicotine synthesis regulation^19^, resistance to biotic and abiotic stress^20^,^21^, influence of tocopherol on leaf senescence^9^, molecular phenotype of lignin-modified tobacco^22^, etc. In this study, the early senescence of tobacco leaves was investigated under natural developmental conditions by using integrated multi-platform metabolomics based on GC-MS, CE-MS and LC-MS. Broad metabolome coverage was achieved, including pigments, lipids, sugars, sterols, amino acids, polyamines and polyphenols. The metabolic changes and related molecular mechanisms in tobacco leaves during early senescence are discussed.

## Results and discussion

### Comprehensive metabolome analysis of tobacco leaves during early senescence

**Figure 1:**
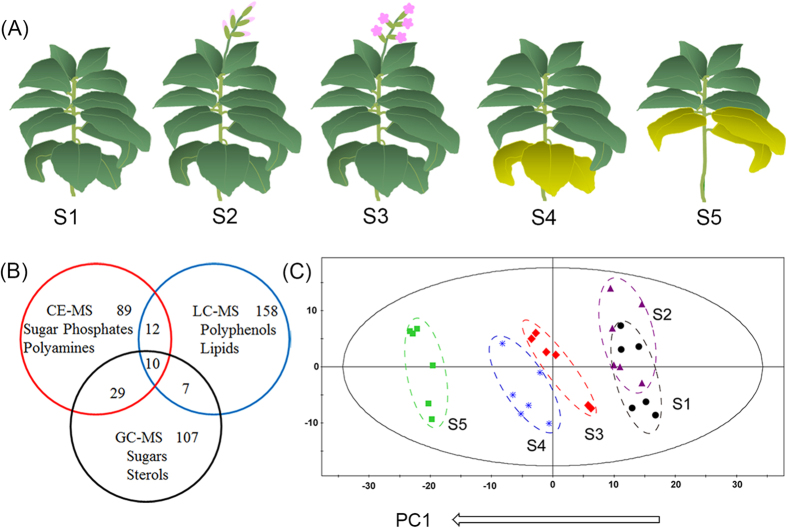
(**A**) Experimental design of tobacco leaves at 5 stages. (**B**) The metabolites detected by CE-MS, GC-MS and LC-MS. The labeled metabolites were specifically detected by each analytical platform. (**C**) PCA score plot of the tobacco leaves at 5 stages. S1, vigorous growth stage. S2, 50% flower bud stage. S3, full-bloom stage. S4, lower leaf ripening stage. S5, middle leaf ripening stage.

Tobacco leaves were picked at the S1, S2, S3, S4 and S5 stages (Fig. 1A) and analyzed by LC-MS, CE-MS and GC-MS. The relative standard deviation (RSD) of each metabolite among quality control (QC) samples was used to evaluate the reproducibility and stability of the three platforms. For the LC-MS analysis, 98% of the lipids and 93% of the relative hydrophilic metabolites were reproducible, with RSDs less than 30%, and the abundance of these metabolites represented 99% and 97% of the total abundance, respectively (Supplementary Fig. S1A,B). For the CE-MS and GC-MS analyses, 97% and 85% metabolites were within 30% of the RSD, respectively, which accounted for 99% and 97% of the total abundance, respectively (Supplementary Fig. S1C,D). These results indicated that the comprehensive metabolomics analysis with these three platforms was robust and reliable.

In total, 190, 140, and 154 metabolites were identified by the LC-MS, CE-MS and GC-MS analyses, respectively (Supplementary Table S1). After removal of the duplicates, 412 metabolites were identified in tobacco leaves (Fig. 1B). For the duplicated data, metabolites with smaller RSDs in the QC samples were reserved for further analysis. Lipids, pigments, polyphenols of phenolic acid esters, flavonoids and coumarin derivatives were detected by LC-MS. Polar metabolites, including amino acids, polyamines, sugars, sugar phosphates, nucleic acids and organic acids, were mainly detected by CE-MS and GC-MS. The metabolites were identified by comparing the MS results with databases (METLIN and the Human Metabolome Database [HMDB] for LC-MS; the National Institute of Standards and Technology [NIST], Wiley and Fiehn for GC-MS; and the Human Metabolome Technologies [HMT] database^23^ for CE-MS) and then confirming the identification with available standards. The lipids and polyphenols were characterized on the basis of their specific fragmentation rules. These metabolites were analyzed with principal component analysis (PCA), as shown in Fig. 1C. The samples were clearly separated into the 5 different stages by principal component 1 (PC1), which accounted for 41% of the total variability. The metabolites were analyzed by nonparametric tests between every two of the five developmental stages, and metabolites with significantly different levels (*p* < 0.05) were chosen for further study.

### Pigments and sugars

**Figure 2:**
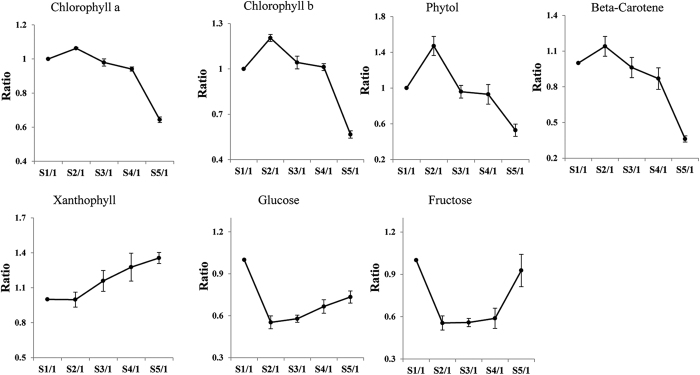
The developmental curves of the chlorophyll a, chlorophyll b, phytol, beta-carotene, glucose and fructose levels. The data points represent the relative contents from S1 to S1, S2 to S1, S3 to S1, S4 to S1 and S5 to S1. The error bars indicate ± SE.

The chloroplast is a very important organelle in plant leaves because it is where photosynthesis occurs. In this study, the levels of chlorophyll a, chlorophyll b, phytol and beta-carotene were significantly decreased from S2 to S5, whereas the xanthophyll content increased after S2 (Fig. 2). The levels of chlorophyll a, chlorophyll b, phytol and beta-carotene decreased 1.7-, 2.1-, 2.8- and 3.2-fold from S2 to S5, respectively. When leaves mature and senesce, the initial phenomena and most marked changes of senescence appear in the chloroplast^6^. The down-regulation of the chlorophyll a and b content indicated that the photosynthetic capacity decreased after S2. Yellow color is an obvious characteristic of leaf senescence. The degradation of chlorophylls and accumulation of xanthophyll may explain the yellowing of the leaves.

Glucose and fructose are important products of photosynthesis and are the most abundant monosaccharides in tobacco leaves. The glucose and fructose levels decreased significantly from S1 to S2 (Fig. 2), earlier than the down-regulation of chlorophyll a and b. Sugars have been reported to regulate the process of leaf senescence^24^. The significant changes in the glucose and fructose levels from S1 to S2 suggested that the leaf senescence might be induced by these sugars before the decrease in photosynthesis.

### Lipid characteristics

#### Glycolipids and triacylglycerols (TGs)

**Figure 3:**
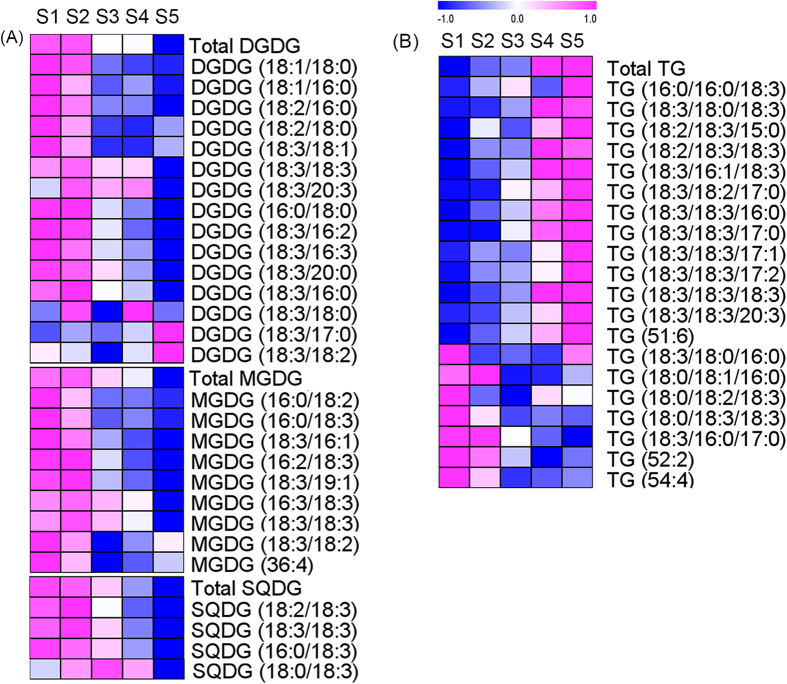
HCA of glycolipids (**A**) and TGs (**B**) at 5 different stages. Red and blue indicate the relative metabolite contents: Red indicates higher contents, and blue indicates lower contents. Abbreviations: MGDG (monogalactosyldiacylglycerol), DGDG (digalactosyldiacylglycerol), SQDG (sulfoquinovosyl diacylglycerol), and TG (triacylglycerol).

Glycolipids including monogalactosyldiacylglycerol (MGDG), digalactosyldiacylglycerol (DGDG) and sulfoquinovosyl diacylglycerol (SQDG) are the main lipids of the thylakoid membrane in chloroplasts. These glycolipids, which are correlated with photosynthesis, are mainly found in oxygen-evolving photosynthetic organisms^25^. Here, the MGDG, DGDG and SQDG levels in tobacco leaves decreased after S2 (Fig. 3A) by 1.8-, 2.0-, and 3.5-fold from S2 to S5, respectively. Typically, because the 16:3 fatty acyl chain is specific for the glycolipids of tobacco leaves, tobacco is a 16:3 plant^26^. The 16:3 chains were abundant in MGDGs and DGDGs. The MGDG (16:3/18:3) and DGDG (16:3/18:3) levels markedly decreased by 3.7- and 8.4-fold from S2 to S5, respectively. The decrease in the levels of these three glycolipids occurred after S2, when the chlorophyll levels began to decrease. Thus, the decrease in the photosynthetic ability occurred at the same time as thylakoid membrane degradation. Moreover, TG is the most common storage lipid in plants. Most TGs were up-regulated (Fig. 3B); the increased TG levels in senescing leaves have been reported to arise from the fatty acid chains released by MGDG and DGDG degradation within chloroplasts^27^,^28^.

#### Sterol lipids and phospholipids

**Figure 4:**
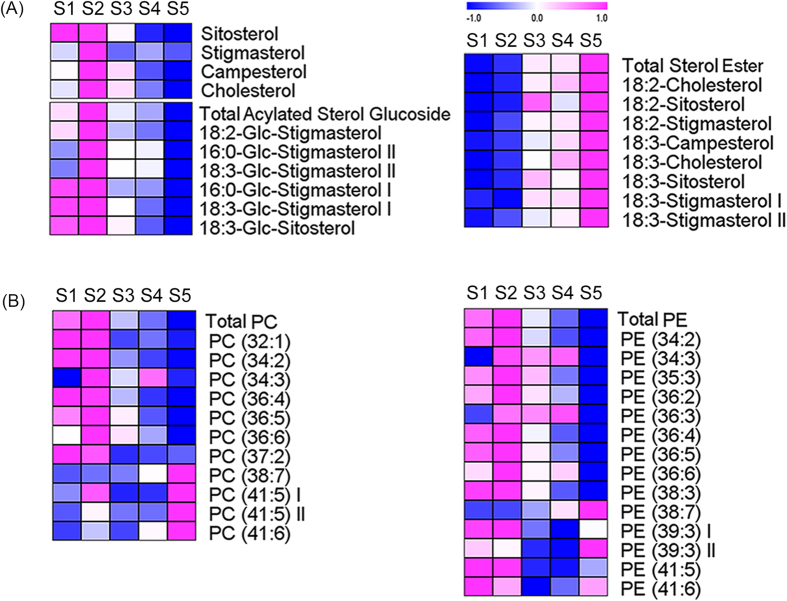
HCA of sterol lipids (**A**) and phospholipids (**B**). Red and blue reflect the relative metabolite contents: Red indicates higher contents, and blue indicates lower contents. Abbreviations: PC (phosphatidylcholine) and PE (phosphatidylethanolamine).

Sterol lipids and phospholipids are abundant in the extraplastidial membranes of plants, including the plasma membrane, tonoplast and endoplasmic reticulum^29^. Stigmasterol, sitosterol and campesterol are the most abundant sterols in plants^30^. Sterols conjugated with fatty acids and glucose were also detected in tobacco leaves. Sterol metabolites showed opposing trends. The free sterol and acylated sterol glucoside contents decreased from S2 to S5, whereas the sterol ester levels increased after S2 (Fig. 4A). The significant changes in the sterol lipids occurred after S2, when the chlorophyll levels began to decrease, thus reflecting their essential role in regulating leaf development and senescence. The stigmasterol, sitosterol and campesterol levels decreased 1.4-, 2.0-, and 1.5-fold from S2 to S5, respectively. The total acylated sterol glucoside levels decreased 2.4-fold from S2 to S5, whereas the total sterol ester levels increased 3.5-fold from S2 to S5. Stigmasterol and sitosterol differ by only a double bond in their side chains, but their effects on the membrane were significantly different. Sitosterol is more efficient than stigmasterol at organizing the fatty acyl chains of membrane phospholipids and controlling the water permeability of the cell membrane^31^,^32^. The ratio of free stigmasterol to sitosterol was up-regulated with the developmental stage (Supplementary Fig. S2A), indicating decreased membrane fluidity and increased water permeability of the cell membrane. The ratio of stigmasterol to sitosterol among the sterol lipids was also calculated. In acylated sterol glucosides, the ratio increased (Supplementary Fig. S2B), whereas in sterol esters, it decreased (Supplementary Fig. S2C). Sterol esters play a crucial role in regulating the concentration of free sterols in the membrane^33^. Enhanced sterol esterification has been reported to be a response to reduced cell proliferation^34^. While the significant increase of sterol esters occurred with the decrease of free sterols at S2, it also implied the occurrence of leaf senescence at S2.

The levels of phospholipids, including phosphatidylcholine (PC) and phosphatidylethanolamine (PE), also showed different developmental trends. The levels of all PCs and PEs decreased from S2 to S4, but the trends deviated from S4 to S5. The levels of phospholipids with shorter fatty acyl chains, such as PC (34:3) and PE (35:3), decreased from S4 to S5, whereas those of phospholipids with longer fatty acyl chains, such as PC (41:5) and PE (39:3), increased from S4 to S5 (Fig. 4B). The total PC and PE levels decreased by 1.7- and 1.5-fold from S2 to S5, respectively. Since phospholipase activity was increased during early senescence^35^, the phospholipid levels decreased. The phospholipid levels decreased slower than the glycolipid levels. When the leaf cells underwent senescence, chloroplast function was first weakened^6^, whereas mitochondrial function was maintained until the last stage of senescence for energy production by respiration^13^. The increase in the levels of phospholipids with longer fatty acyl chains from S4 to S5 indicated that membrane fluidity decreased during leaf senescence.

### Changes in amino acids, polyamines and alkaloids

**Figure 5:**
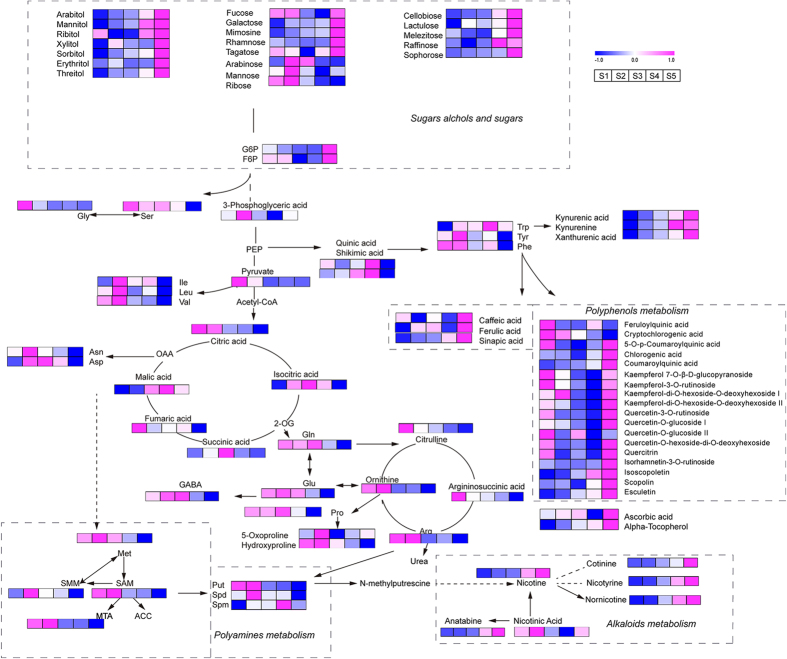
Metabolic pathways of the polar metabolites. The metabolic pathways include sugar and sugar alcohol metabolism, amino acid metabolism, polyamine metabolism, alkaloid metabolism and polyphenol metabolism. Red and blue reflect the relative metabolite contents: Red indicates higher contents, and blue indicates lower contents. Abbreviations: G6P (glucose 6-phosphate), F6P (fructose 6-phosphate), PEP (phosphoenolpyruvic acid), OAA (oxaloacetate), 2-OG (2-oxoglutaric acid), Glu (glutamic acid), Gln (glutamine), Asp (aspartic acid), Asn (asparagine), Gly (glycine), Ser (serine), Ile (isoleucine), Val (valine), Leu (leucine), Trp (tryptophan), Tyr (tyrosine), Phe (phenylalanine), Met (methionine), SAM (S-adenosylmethionine), SMM (S-methylmethionine), MTA (5-methylthioadenosine), and ACC (1-aminocyclo-propane-1-carboxylic acid).

Nitrogen utilization is an important physiological activity in plant growth and development. Amino acid metabolism is the main component of nitrogen metabolism. The total amino acid content decreased 2.6-fold from S2 to S5, reflecting the lower levels of nitrogen assimilation after S2 (Supplementary Fig. S3A). Nitrogen assimilation is highly coordinated with photosynthesis^36^. Because the chlorophyll a and b levels decreased after S2, the decrease in photosynthesis and the level of nitrogen assimilation proceeded synergistically during leaf senescence. The levels of glutamic acid (Glu) and aspartic acid (Asp), which are precursors of many other amino acids^37^, decreased 1.9- and 2.9-fold from S2 to S5, respectively (Fig. 5). Glutamine (Gln) and asparagine (Asn), the main N-rich amino acids in leaves, are predominantly involved in fixing inorganic nitrogen^38^, and their levels decreased 4.0- and 5.5-fold, respectively; additionally, the Gln/total amino acid and Asn/total amino acid ratios decreased (Supplementary Fig. S3B,C). Thus, N was translocated to the young upper leaves during leaf early senescence. In *Arabidopsis thaliana*, much lower nitrogen levels were observed in senescing leaves than in pre-senescence leaves^5^, and nitrogen remobilizes from senescing leaves to storage organs or young expanding leaves^39^. Glycine (Gly) and serine (Ser) are two essential amino acids formed during photorespiration, and the Gly/Ser ratio reflects the rate of photorespiration^40^. The Gly and Ser levels decreased during early senescence (Fig. 5), and the Gly/Ser ratio decreased from S1 to S3 and increased after S3 (Supplementary Fig. S3D). The increase in the ratio reflected the increase in photorespiration after S3.

Polyamines are specific growth regulators with crucial physiological functions in fundamental cellular processes, including cell division, differentiation, proliferation and senescence^17^,^41^. Polyamine metabolism also participates in nitrogen metabolism. Putrescine, spermidine and spermine are the main diamine, triamine and tetramine in plants, respectively. Putrescine is synthesized from arginine and ornithine by arginine and ornithine decarboxylase and is then transformed to spermidine and spermine through the respective aminopropyl transferases^42^. S-adenosylmethionine (SAM) plays a vital role in physiological process, and decarboxylated SAM acts as an aminopropyl donor^43^. SAM connects methionine (Met) and polyamine metabolism and is metabolized to 1-aminocyclo-propane-1-carboxylic acid (ACC), S-methylmethionine (SMM) and 5-methylthioadenosine (MTA). The SAM, ACC, SMM, MTA and Met levels decreased after S2. The putrescine and spermidine levels also decreased after S2, whereas the spermine levels increased from S2 to S4 and decreased after S4 (Fig. 5). Polyamines are correlated with the stabilization of photosynthetic apparatus by binding to photosynthetic complexes, and spermine is more effective than putrescine and spermidine^41^,^44^. The decrease in the putrescine and spermidine levels and the increase in the spermine levels from S2 to S4 indicated that spermine effectively protected the photosystem. The levels of these three polyamines all decreased from S4 to S5, thus suggesting the acceleration of leaf senescence.

Alkaloids are a class of secondary metabolites that contain nitrogen. Nicotine is the most abundant alkaloid in tobacco, which is synthesized in tobacco roots, transported to the leaves and stored in vacuoles of leaves^45^. In the study, the alkaloids including nicotine, nicotinic acid, anatabine, cotinine, nicotyrine and nornicotine up-regulated with the development stages (Fig. 5). The content of nicotine showed distinct enhancement after S3 and increased for 2.5-fold from S3 to S5. Nornicotine is a secondary alkaloid and is formed through the oxidative N-demethylation of nicotine^46^. The level of nornicotine was found obvious accumulation after S2 and increased for 4.6-fold from S2 to S5. The increase of nornicotine was earlier than the nicotine. The conversion of nicotine to nornicotine was mostly found in senescing leaves^47^,^48^. Then these results could reflect the onset of leaf senescence after S2 and the accumulation of alkaloids during leaf early senescence.

### Osmoprotectants and antioxidants

Sugar polyols are reduced forms of aldose and ketose sugars. Sugar polyols have been reported to act as osmoprotectants by increasing the hydration around macromolecules through their water-like hydroxyl groups^18^,^49^. As shown in Fig. 5, ribitol, xylitol, mannitol, arabitol, sorbitol, threitol and erythritol were detected in tobacco leaves and were up-regulated from S4 to S5. The accumulation of sugar polyols may help to maintain the cell hydration level and cellular functions during leaf senescence. In addition to sugar polyols, hexose monosaccharides, including galactose, rhamnose, tagatose, and fucose, and polysaccharides, including raffinose, melezitose, lactulose, cellobiose, and sophorose, were also up-regulated during the final stages of development.

Polyphenols include phenolic acid esters, flavonoids and coumarin derivatives. The following phenolic acid esters were detected: esters of coumaric acid, caffeic acid, ferulic acid and quinic acid. Flavonoids include the glycosides of kaempferol and quercetin. The coumarin derivatives detected include esculetin, isoscopoletin and scopolin. Most of the polyphenols were up-regulated from S4 to S5 (Fig. 5). The phenolic acids-caffeic acid, ferulic acid and sinapic acid-were also up-regulated from S4 to S5. Polyphenols originate from phenylalanine and are synthesized by the phenylpropanoid pathway. The accumulation of polyphenols resulted in phenylalanine consumption because the phenylalanine levels were significantly decreased from S4 to S5. Phenolic acid esters and flavonoids have been reported to be antioxidants that scavenge free radicals^50^. Numerous reactive oxygen species and radicals are produced during senescence^51^, inducing the synthesis of phenolic acid esters and flavonoids. Ascorbic acid and alpha-tocopherol are important antioxidants and were also significantly up-regulated from S4 to S5. These results indicated that the antioxidant system was activated during leaf senescence.

## Conclusions

An integrated multi-platform metabolomics analysis using GC-MS, CE-MS and LC-MS was developed to investigate natural early tobacco leaf senescence. Broad metabolome coverage was achieved, and 412 primary and secondary metabolites were identified. Comprehensive time-dependent metabolomics investigation was performed on tobacco middle leaves at 5 developmental stages (vigorous growth, 50% flower bud, full-bloom, lower leaf ripening, and middle leaf ripening). The multivariate analysis illustrated that the metabolic profiles of the tobacco leaves were strongly influenced by their developmental stages. With the onset of senescence, significant physiological variations occurred that activated or inhibited various metabolic pathways. Senescence-related loss of photosynthetic capacity was observed after the 50% flower bud stage, as determined by chloroplast thylakoid membrane degradation and a rapid decrease in the chlorophyll levels. Extraplastidial membrane degradation and decreased membrane fluidity were noted during leaf senescence. Leaf nutrient remobilization was observed as an accumulation of storage lipids and a decrease in free amino acid levels. The conversion of nicotine to nornicotine occurred. The antioxidant system was activated at the lower leaf ripening stage. The overall metabolite changes along the development stages were shown in supplementary Fig. S4. These results revealed the metabolic changes and molecular mechanisms during early senescence in tobacco leaves. Further investigations needs be performed to obtain a deeper understanding of the metabolic mechanisms.

## Methods

### Reagents and chemicals

Gradient elution solvents, including high-performance LC (HPLC)-grade acetonitrile and isopropanol, were purchased from Merck (Darmstadt, Germany). The HPLC-grade extraction solvents methyl tert-butyl ether (MTBE), chloroform and methanol (MeOH) were purchased from Sigma-Aldrich (St. Louis, MO, USA) and Merck (Darmstadt, Germany). HPLC-grade dichloromethane (CH_2_Cl_2_) was purchased from Merck (Darmstadt, Germany). Ammonium acetate, methoxyamine hydrochloride, N-methyl-N-(trimethylsilyl)-trifluoroacetamide (MSTFA) and pyridine were purchased from Sigma-Aldrich (St. Louis, MO, USA). Ultrapure water was acquired from a Milli-Q water system (Millipore, Billerica, USA). Internal standards were purchased from Sigma-Aldrich (St. Louis, MO, USA), J&K chemicals (Shanghai, China) and Wako Pure Chemical Industries (Japan). Lipid standards were purchased from Avanti Polar Lipids Inc. (Alabaster, AL, USA).

### Sample information

The plants of *Hongda* flue-cured tobacco were grown in an open field in Guizhou, China, which is an appropriate growth environment for them, in 2014. Tobacco leaves were picked at 5 stages: 34 days (S1), 51 days (S2), 67 days (S3), 76 days (S4) and 95 days (S5) after transplanting. At S1, tobacco was in the vigorous growth stage. At S2, 50% of the flowers were budding. At S3, all the flowers were opened. At S4, the lower leaves ripened and were harvested. At S5, the middle leaves ripened and turned yellow. The tenth leaves (middle leaves) from the bottom were picked from the plants at each stage, and six biological replicates were collected. The harvested tobacco leaves were immediately placed in liquid nitrogen to avoid changes in the metabolites because of enzyme activity. Then, the fresh leaves were ground into a powder and freeze-dried. Equal amounts of all samples were mixed to provide QC samples, which were equally inserted in the analytical batch to monitor the analytical performance.

### LC-MS untargeted metabolomics analysis and lipidomic analysis

The LC-MS method has been described previously^15^. Ten milligrams of leaf power was weighed into a 2-mL Eppendorf tube. Then, 370 μL of MeOH, 450 μL of MTBE and 680 μL of H_2_O were added. After vortexing and centrifuging, two clear phases were obtained, and a precipitate formed at the bottom of the tube. The green upper fraction contained the liposoluble compounds in MTBE. Then, 200 μL was taken out for lipidomic analysis. The underlayer contained the relative hydrophilic metabolites, and a 500-μL aliquot was collected for the untargeted metabolomics analysis. Lyso PE (14:0), lyso PC (19:0), PC (14:0/14:0), diacylglycerol (DG) (12:0/12:0), TG (17:0/17:0/17:0), vietexin, L-tryptophan-d5, L-phenyl-d5-alanine and decanoyl carnitine-S3 in MeOH were added as internal standards. The LC-MS untargeted metabolomics analysis was performed on an Agilent 1200 rapid resolution LC with Agilent 6510 electrospray ionization quadrupole time-of-flight MS (Agilent, Santa Clara, CA, USA). For the lipidomic analysis, a T3 column (1.8-μm particle size, 2.1 × 100 mm, Waters Acquity UPLC HSS, Ireland) was used for the separation with a column temperature of 55 °C and a flow rate of 0.26 mL/min. Mobile phase A was acetonitrile and water (3:2, v/v) with 10-mM ammonium acetate. Mobile phase B was isopropanol and water (9:1, v/v) with 10-mM ammonium acetate. A C18 column was used for the separation (1.8-μm particle size, 2.1 × 100 mm, Agilent ZORBAX SB-AQ, USA) of the relative hydrophilic metabolites with a column temperature of 50 °C and a flow rate of 0.30 mL/min. Mobile phases A and B were composed of water and acetonitrile with 0.1% formic acid, respectively. For MS, a gas temperature of 350 °C and a drying gas flow rate of 9 L/min were used. The voltages applied to the entrance of the capillary (Vcap), fragmentor, skimmer and octopole were 4,000 V, 175 V, 65 V and 750 V, respectively. The data were acquired in positive mode.

### CE-MS untargeted analysis

This method has been described previously^23^. First, 13 mg of tobacco powder was added to 1.8 mL of extraction solvent containing methanol, chloroform and water (1:1:4, v/v/v). The internal standards were L-methionine sulfone and D-camphor-10-sulfonic acid sodium salt (10 mM) dissolved in methanol. After vortexing and centrifuging, a 400-μL aliquot of the aqueous phase was transferred to a 5-kDa-cutoff filter (Millipore). The sample was ultra-filtered at 12,000 g for 2 h and then freeze-dried. The dried sample was dissolved in water containing 3-aminopyrrolidine dihydrochloride, N, N-diethyl-2-phenylacetamide, trimesic acid and 2-naphthol-3, 6-disulfonic acid disodium salt (1 mM). The analysis was performed by using an Agilent G7100A CE with an Agilent G6224A electrospray ionization time-of-flight MS (Agilent, Santa Clara, CA, USA). A fused-silica capillary (i.d. 50 μm × 80 cm) was obtained from Human Metabolome Technologies, Inc. (HMT, Japan). The capillary temperature was set at 20 °C. The sheath liquid was methanol/water (1:1, v/v) and 0.1-μM hexakis (2,2-difluoroethoxy) phosphazene, and the flow rate was 10 μL/min. The following cation-mode conditions were used: water with 1-M formic acid was used as the background electrolyte; the sample was injected at a pressure of 50 mbar for 10 s and analyzed at 27 kV; the gas temperature was 300 °C; the drying gas flow was 7 L/min; and the Vcap, fragmentor, skimmer, and octopole voltages were 4,000 V, 114 V, 50 V, and 625 V, respectively. The following anion mode conditions were used: water with 50-mM ammonium acetate (pH 8.5) was used as the background electrolyte; the sample was injected at a pressure of 50 mbar for 25 s and analyzed at 30 kV; and the Vcap and fragmentor voltages were 3,500 V and 125 V, respectively.

### GC-MS pseudo-targeted analysis

GC-MS analysis has been described in detail previously^16^. First, 10 mg of tobacco leaves was extracted with 1.5 mL of isopropanol, acetonitrile and water (3:3:2, v/v/v). Tridecanoic acid was used as the internal standard. After vortexing and centrifuging, a 500-μL aliquot of the supernatant was removed and freeze-dried. The oximation reaction was performed by adding 100 μL of the methoxyamine solution to the dried sample and incubating the mixture in a 37 °C water bath for 90 min. Subsequently, 80 μL of MSTFA was added to the sample for silylation reaction and incubated in a 37 °C water bath for 60 min. The sample was analyzed on a Shimadzu single quadrupole GCMS-QP2010 plus (Kyoto, Japan). An Agilent DB-5 MS fused silica capillary column (30 m × 0.25 mm × 0.25 μm) was used for separation. Selected ion monitoring (SIM) mode was chosen for the instrumental analysis. Peak deconvolution and the identification and selection of the characteristic ions were performed by using AMDIS, Leco ChromaTOF and in-house software. The spit ratio was 10:1. The carrier gas was helium, and the flow rate was 1.2 mL/min. The injector, interface and ion source temperatures were set to 280 °C, 280 °C, and 240 °C, respectively. The detector voltage was 0.92 kV. The ionization mode was electron impact (EI) at 70 eV.

### Data processing

For LC-MS, peak alignment was achieved with XCMS^52^. For CE-MS, peak deconvolution, detection and area integration were performed by using Agilent Masshunter qualitative and quantitative analysis software. For GC-MS, the molecular features were obtained from the acquired SIM tables. Peak tables with mass, retention time and abundance were established for the three methods. PCA was performed to investigate the changes among the different developmental stages by using SIMCA-P software (Version 11.5, Umetrics AB, Umeå, Sweden). Nonparametric tests were conducted to identify the senescence-related metabolites with Multiexperiment Viewer (MeV) (Version 4.9, Dana-Farber Cancer Institute, Boston, MA, USA). The significance level was less than 0.05. Hierarchical cluster analysis (HCA) was also performed with MeV software.

## Additional Information

**How to cite this article**: Li, L. *et al*. Comprehensive investigation of tobacco leaves during natural early senescence via multi-platform metabolomics analyses. *Sci. Rep.* **6**, 37976; doi: 10.1038/srep37976 (2016).

**Publisher's note:** Springer Nature remains neutral with regard to jurisdictional claims in published maps and institutional affiliations.

## Supplementary Material

Supplementary Information
