# Supplementary material for: Comprehensive investigation of tobacco leaves during natural early senescence via multi-platform metabolomics analyses
Source: Sci Rep. 2016 Nov 29;6:37976. doi: 10.1038/srep37976 (PMC5126694; doi:10.1038/srep37976)
Supplement: Supplementary Information [file srep37976-s1.pdf]

Supplementary Information

Comprehensive investigation of tobacco leaves during natural early senescence via multi-platform metabolomics analyses

Lili Li[1,2,], Jieyu Zhao[1,2,], Yanni Zhao[1,2], Xin Lu[1,2*], Zhihui Zhou[1,2] , Chunxia Zhao[1,2*], Guowang Xu[1,2]

1, Key Laboratory of Separation Science for Analytical Chemistry, Dalian Institute of Chemical Physics, Chinese Academy of Sciences, Dalian 116023, China

2, University of Chinese Academy of Sciences, Beijing, 100049, China

***** Address correspondence to:

Prof. Dr. Xin Lu, Key Laboratory of Separation Science for Analytical Chemistry, Dalian Institute of Chemical Physics, Chinese Academy of Sciences, Dalian 116023, China. Tel.: 0086-411-84379532. E-mail: luxin001@dicp.ac.cn

Dr. Chunxia Zhao, Key Laboratory of Separation Science for Analytical Chemistry, Dalian Institute of Chemical Physics, Chinese Academy of Sciences, Dalian 116023, China. Tel.: 0086-411-84379757. E-mail: zhaocx@dicp.ac.cn

Number of supplementary figures: 4

Number of supplementary tables: 1

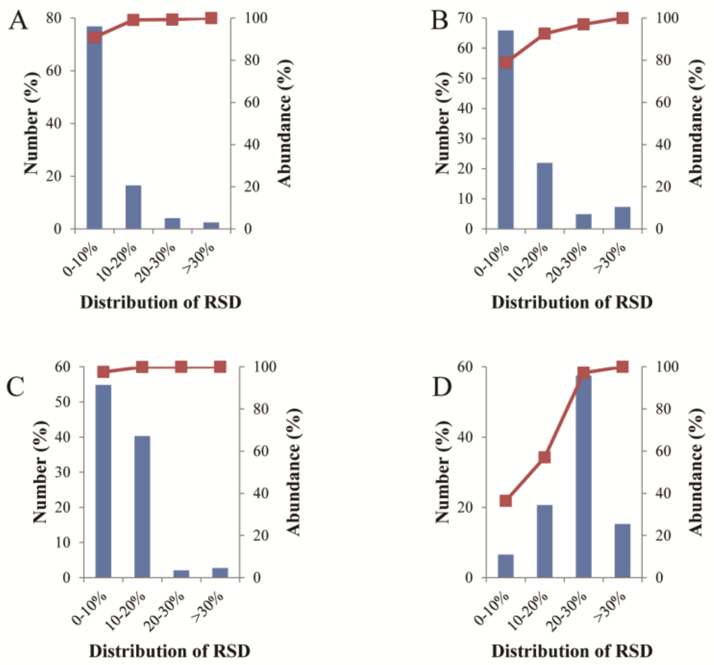

Supplementary Figure S1. RSD distributions for the LC-MS lipid analysis (A), LC-MS relative hydrophilic metabolite analysis (B), CE-MS (C) and GC-MS (D).

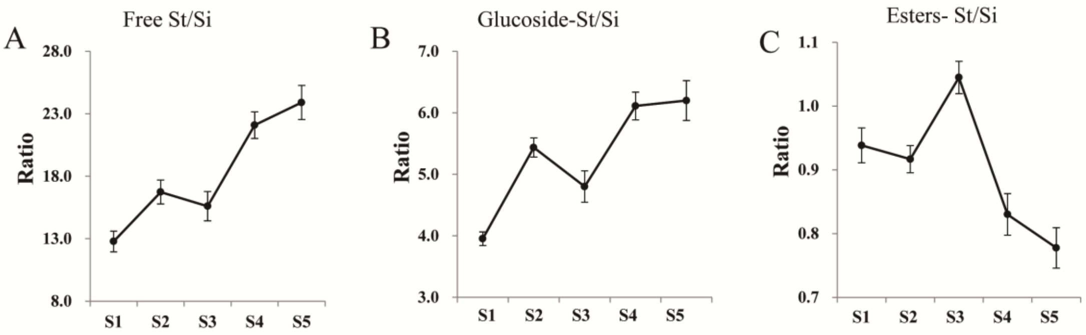

Supplementary Figure S2. Ratios of stigmasterol (St) and sitosterol (Si) in their free forms (A), acylated sterol glucosides (B) and sterol esters (C). Each data point represents mean ± SE.

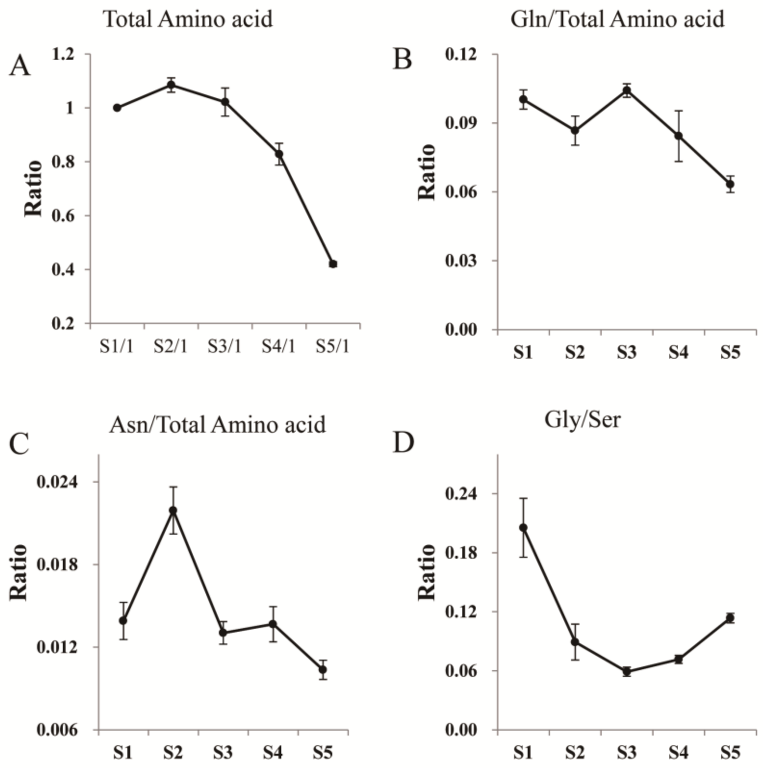

Supplementary Figure S3. A, The developmental curve of the total amino acid contents. The data points were the relative contents from S1 to S1, S2 to S1, S3 to S1, S4 to S1 and S5 to S1. Gln/total amino acids (B), Asn/Total amino acids (C) and Gly/Ser ratios are presented. The error bars represent ±SE.

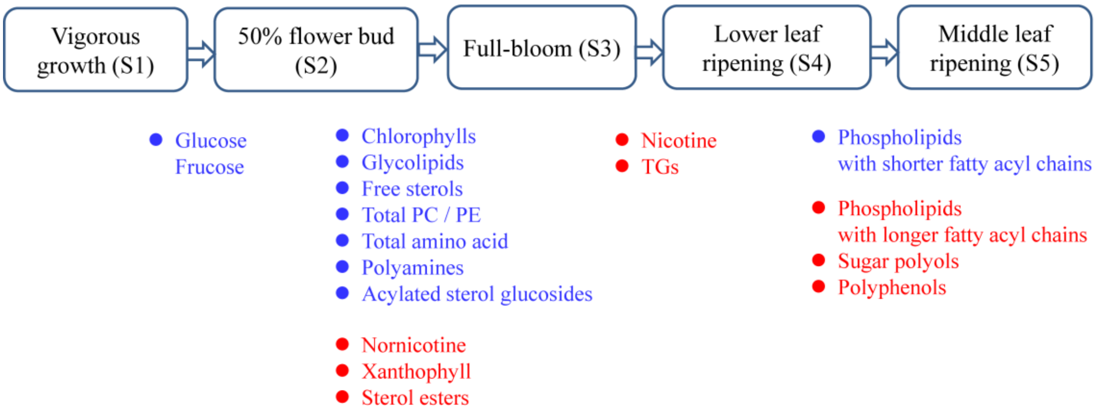

Supplementary Figure S4. The overall metabolomics changes in tobacco leaves along with the 5 development stages. The blue color represents that the contents of these metabolites down-regulated. The red color represents that the contents of these metabolites up-regulated.

Supplementary Table S1. The identified metabolites in tobacco leaves by GC-MS, LC-MS and CE-MS. The LC/CE-MS, GC/CE-MS and LC/GC-MS showed that the metabolite was detected by two platforms. The LC/GC/CE-MS showed that the metabolite was detected by three platforms.

Compounds	Platforms	Compounds	Platforms
Betaine	LC/CE-MS	Nicotyrine	GC-MS
Choline	LC/CE-MS	Nonadecanoic acid	GC-MS
Nicotinamide	LC/CE-MS	Nonanoic acid	GC-MS
Spermidine	LC/CE-MS	Oleic acid	GC-MS
Spermine	LC/CE-MS	Oxalic acid	GC-MS
MTA	LC/CE-MS	Phenol	GC-MS
Adenine	LC/CE-MS	Phosphoric acid	GC-MS
Adenosine	LC/CE-MS	p-Hydroxybenzoic acid	GC-MS
Guanine	LC/CE-MS	Phytol	GC-MS
Guanosine	LC/CE-MS	Porphine	GC-MS
Riboflavin	LC/CE-MS	Propenoic acid	GC-MS
Glutathione	LC/CE-MS	Pyrogallol	GC-MS
Anatabine*	LC/GC-MS	Pyruvic acid	GC-MS
Cotinine	LC/GC-MS	Raffinose	GC-MS
Nornicotine	LC/GC-MS	Rhamnose1	GC-MS
Phytosphingosine	LC/GC-MS	Ribitol	GC-MS
Palmitic acid	LC/GC-MS	Ribonic acid-gamma-lactone	GC-MS
Caffeic acid	LC/GC-MS	Ribose	GC-MS
Chlorogenic acid	LC/GC-MS	Sitosterol	GC-MS
Glycerol 3-phosphate	GC/CE-MS	Sophorose	GC-MS
Urea	GC/CE-MS	Sorbitol	GC-MS
Putrescine	GC/CE-MS	Sphingosine	GC-MS
5-Aminovaleric acid	GC/CE-MS	Stearic acid	GC-MS
5-Oxoproline	GC/CE-MS	Stigmasterol	GC-MS
Ala	GC/CE-MS	Succinic acid	GC-MS
Asn	GC/CE-MS	Sucrose	GC-MS
Gly	GC/CE-MS	Tagatose	GC-MS
Ile	GC/CE-MS	Threitol	GC-MS
Leu	GC/CE-MS	Threonic acid	GC-MS
Lys	GC/CE-MS	Threose	GC-MS
Met	GC/CE-MS	trans-4-hydroxy-L-proline	GC-MS
Ser	GC/CE-MS	Trehalose	GC-MS
Thr	GC/CE-MS	Xylitol	GC-MS
Val	GC/CE-MS	Xylose	GC-MS
2-Isopropylmalic acid	GC/CE-MS	δ-Amyrenol	GC-MS
3-Hydroxy-3-methylglutaric acid	GC/CE-MS	1,2,4-BUTANTRIOl	GC-MS
4-Guanidinobutyric acid	GC/CE-MS	2,2-BIPYRIDINE	GC-MS
Citramalic acid	GC/CE-MS	2,3-dihydroxypyridine	GC-MS
Citric acid	GC/CE-MS	2-Hydroxycaproic acid	GC-MS

Fumaric acid	GC/CE-MS	2-Hydroxyglutaric acid	GC-MS
Glyceric acid	GC/CE-MS	2-Hydroxypyridine	GC-MS
Isocitric acid	GC/CE-MS	2-Keto-l-gluconic acid	GC-MS
Lactic acid	GC/CE-MS	3-hydroxypyridine	GC-MS
Malic acid	GC/CE-MS	4-hydroxybenzoic acid	GC-MS
Mucic acid	GC/CE-MS	4-hydroxybutyric acid	GC-MS
Quinic acid	GC/CE-MS	4-Hydroxycinnamic acid	GC-MS
Shikimic acid	GC/CE-MS	4-Hydroxyphenylpyruvic acid	GC-MS
Gluconic acid	GC/CE-MS	4-hydroxypyridine	GC-MS
Trigonelline	LC/GC/CE-MS	Beta-Carotene	LC-MS
Gln	LC/GC/CE-MS	Caffeoyl putrescine I	LC-MS
Glu	LC/GC/CE-MS	Caffeoyl putrescine II	LC-MS
Asp	LC/GC/CE-MS	Caffeoyl spermidine I	LC-MS
Phe	LC/GC/CE-MS	Caffeoyl spermidine II	LC-MS
Pro	LC/GC/CE-MS	Chlorophyll a	LC-MS
Trp	LC/GC/CE-MS	Chlorophyll b	LC-MS
Tyr	LC/GC/CE-MS	Coumaroyl putrescine	LC-MS
Uracil	LC/GC/CE-MS	Coumaroylquinic acid	LC-MS
Pantothenic acid	LC/GC/CE-MS	Cryptochlorogenic acid	LC-MS
ACC	CE-MS	Esculetin	LC-MS
ADP	CE-MS	Feruloyl putrescine I	LC-MS
Adrenaline	CE-MS	Feruloyl putrescine II	LC-MS
Ala-Ala	CE-MS	Feruloyl quinic acid	LC-MS
Arg	CE-MS	Feruloyl spermidine I	LC-MS
Argininosuccinic acid	CE-MS	Feruloyl spermidine II	LC-MS
Ascorbic acid	CE-MS	Isorhamnetin-3-O-rutinoside	LC-MS
ATP	CE-MS	Isoscopoletin	LC-MS
Betaine aldehyde	CE-MS	Kaempferol 7-O-β-D-glucopyranoside	LC-MS
Carnitine	CE-MS	Kaempferol-3-O-rutinoside	LC-MS
Citrulline	CE-MS	Kaempferol-O-hexoside-hexoside-deoxyhexoside I	LC-MS
Cytidine	CE-MS	Kaempferol-O-hexoside-hexoside-deoxyhexoside II	LC-MS
Cytosine	CE-MS	Kaempferol--O-rutinoside	LC-MS
Dopamine	CE-MS	Kynurenic acid	LC-MS
Ethanolamine phosphate	CE-MS	Nicotinic acid	LC-MS
Fructose 6-phosphate	CE-MS	Oleamide	LC-MS
GABA	CE-MS	Quercetin-3-O-rutinside	LC-MS
Glucaric acid	CE-MS	Quercetin-O-glucoside I	LC-MS
Glucose 1-phosphate	CE-MS	Quercetin-O-glucoside II	LC-MS
Glucose 6-phosphate	CE-MS	Quercetin-O-hexoside-deoxyhexoside-deoxyhexoside	LC-MS
Glucuronic acid	CE-MS	Quercetin-O-rutinside	LC-MS

Glutathione (GSSG)	CE-MS	Quercitrin	LC-MS
Glycerophosphocholine	CE-MS	Scopoletin	LC-MS
Gly-Leu	CE-MS	Scopolin	LC-MS
His	CE-MS	Hydroxy-4-(3-pyridyl)-butanoic acid	LC-MS
Hydroxyproline	CE-MS	hydroxy-tridecanoic acid	LC-MS
Imidazole-4-acetic acid	CE-MS	Xanthophyll	LC-MS
Inosine	CE-MS	Xanthurenic acid	LC-MS
Isoamylamine	CE-MS	3-Formylindole	LC-MS
Isobutylamine	CE-MS	4-O-p-Coumaroylquinic acid	LC-MS
Isopropanolamine	CE-MS	5-O-p-Coumaroylquinic acid	LC-MS
Kynurenine	CE-MS	14:0-Campesterol	LC-MS
Methionine sulfoxide	CE-MS	16:0-Glc-Stigmasterol I	LC-MS
N5-Ethylglutamine	CE-MS	16:0-Glc-Stigmasterol II	LC-MS
N6,N6,N6-Trimethyllysine	CE-MS	18:2-Cholesterol	LC-MS
N8-Acetylspermidine	CE-MS	18:2-Glc-Stigmasterol	LC-MS
N-Acetylglucosamine	CE-MS	18:2-Sitosterol	LC-MS
N-Acetylmuramic acid	CE-MS	18:2-Stigmasterol	LC-MS
N-Acetylputrescine	CE-MS	18:3-Campesterol	LC-MS
NAD+	CE-MS	18:3-Cholesterol	LC-MS
NADP+	CE-MS	18:3-Glc-Sitosterol	LC-MS
N-Formylmethionine	CE-MS	18:3-Glc-Stigmasterol I	LC-MS
O-Acetylcarnitine	CE-MS	18:3-Glc-Stigmasterol II	LC-MS
O-Acetylserine	CE-MS	18:3-Sitosterol	LC-MS
Ornithine	CE-MS	18:3-Stigmasterol I	LC-MS
Phenylpyruvic acid	CE-MS	18:3-Stigmasterol II	LC-MS
Phosphorylcholine	CE-MS	d18:0-C28:3-Glc-Ceramide	LC-MS
Pipecolic acid	CE-MS	d18:2-C16:0h-Glc-Ceramide	LC-MS
Piperidine	CE-MS	d18:2-C16:1 ceramide	LC-MS
Prostaglandin F2α	CE-MS	d18:2-C16:1-Glc-Ceramide	LC-MS
Pyridoxal	CE-MS	DG (18:0/18:3)	LC-MS
Pyridoxamine	CE-MS	DG (18:2/18:3)	LC-MS
Pyridoxamine 5'-phosphate	CE-MS	DG (18:3/16:0)	LC-MS
Pyridoxine	CE-MS	DG (18:3/18:3)	LC-MS
S-Adenosylhomocysteine	CE-MS	DGDG (16:0/18:0)	LC-MS
S-Adenosylmethionine	CE-MS	DGDG (18:1/16:0)	LC-MS
Sedoheptulose 7-phosphate	CE-MS	DGDG (18:1/18:0)	LC-MS
Sinapic acid	CE-MS	DGDG (18:2/16:0)	LC-MS
S-Methylmethionine	CE-MS	DGDG (18:2/18:0)	LC-MS
Thiamine	CE-MS	DGDG (18:3/16:0)	LC-MS
Thiamine phosphate	CE-MS	DGDG (18:3/16:2）	LC-MS
Threonic acid	CE-MS	DGDG (18:3/16:3)	LC-MS
Trimethylamine N-oxide	CE-MS	DGDG (18:3/17:0) I	LC-MS
Tropine	CE-MS	DGDG (18:3/17:0) II	LC-MS

Tryptamine	CE-MS	DGDG (18:3/18:0)	LC-MS
Tyramine	CE-MS	DGDG (18:3/18:1)	LC-MS
UDP	CE-MS	DGDG (18:3/18:2)	LC-MS
UDP-glucose	CE-MS	DGDG (18:3/18:3)	LC-MS
UDP-N-acetylglucosamine	CE-MS	DGDG (18:3/20:0)	LC-MS
Uric acid	CE-MS	DGDG (18:3/20:3)	LC-MS
Uridine	CE-MS	lysoPC (16:0)	LC-MS
Urocanic acid	CE-MS	MGDG (16:0/18:2)	LC-MS
UTP	CE-MS	MGDG (16:0/18:3)	LC-MS
Z-Gly	CE-MS	MGDG (16:2/18:3)	LC-MS
β-Ala	CE-MS	MGDG (16:3/18:3)	LC-MS
γ-Glu-Cys	CE-MS	MGDG (18:3/16:1)	LC-MS
1-Aminocyclopentanecarboxylic acid	CE-MS	MGDG (18:3/18:2)	LC-MS
1-Methyladenosine	CE-MS	MGDG (18:3/18:3)	LC-MS
1-Phenylethylamine	CE-MS	MGDG (18:3/19:1)	LC-MS
2-Aminoadipic acid	CE-MS	MGDG (36:4)	LC-MS
2-Aminoisobutyric acid	CE-MS	PC (32:0)	LC-MS
2-Methylserine	CE-MS	PC (32:1)	LC-MS
3-Hydroxybutyric acid	CE-MS	PC (34:1)	LC-MS
3-Hydroxykynurenine	CE-MS	PC (34:2)	LC-MS
3-Methyladenine	CE-MS	PC (34:3)	LC-MS
3-Phosphoglyceric acid	CE-MS	PC (36:3)	LC-MS
5-Amino-4-oxovaleric acid	CE-MS	PC (36:4)	LC-MS
5-Methylcytosine	CE-MS	PC (36:5)	LC-MS
6-Aminohexanoic acid	CE-MS	PC (36:6)	LC-MS
Acetol	GC-MS	PC (37:2)	LC-MS
Acetophenone	GC-MS	PC (38:7)	LC-MS
Alpha Ketoglutaric acid	GC-MS	PC (39:3)	LC-MS
Alpha-Tocopherol	GC-MS	PC (41:5) I	LC-MS
Arabinose	GC-MS	PC (41:5) II	LC-MS
Arabitol	GC-MS	PC (41:6)	LC-MS
Arachidic acid	GC-MS	PE (34:2)	LC-MS
Arbutin	GC-MS	PE (34:3)	LC-MS
Ascorbic acid	GC-MS	PE (35:3) I	LC-MS
Azelaic acid	GC-MS	PE (35:3) II	LC-MS
Benzoic acid	GC-MS	PE (36:2)	LC-MS
Campesterol	GC-MS	PE (36:3)	LC-MS
Capric acid	GC-MS	PE (36:4)	LC-MS
Cellobiose	GC-MS	PE (36:5)	LC-MS
Cholesterol	GC-MS	PE (36:6)	LC-MS
cis-10-Nonadecenoic acid, trimethylsilyl ester	GC-MS	PE (38:3)	LC-MS
Coniferyl alcohol	GC-MS	PE (38:7)	LC-MS

Cys	GC-MS	PE (39:3) I	LC-MS
Dehydroascorbic acid	GC-MS	PE (39:3) II	LC-MS
D-glucose-6-phosphate	GC-MS	PE (41:5) I	LC-MS
Dodecanoic acid, 7,11-dimethyl-3-trimethylsilyloxy-3- trimethylsilyloxymethyl-, trimethylsilyl ester	GC-MS	PE (41:5) II	LC-MS
DOPA	GC-MS	PE (41:6) I	LC-MS
Dopamine	GC-MS	PE (41:6) II	LC-MS
Erythritol	GC-MS	SQDG (16:0/18:3)	LC-MS
Ethanolamine	GC-MS	SQDG (18:0/18:3)	LC-MS
Ethylene glycol	GC-MS	SQDG (18:2/18:3)	LC-MS
Ferulic acid	GC-MS	SQDG (18:3/18:3)	LC-MS
Fructose	GC-MS	t18:1-C23:3-Glc-Ceramide	LC-MS
Fucose	GC-MS	t18:1-C24:0h-Glc-Ceramide	LC-MS
Galactinol	GC-MS	t18:1-C24:1-Glc-Ceramide	LC-MS
Galactinol	GC-MS	t18:1-C25:0h-Glc-Ceramide	LC-MS
Galactose	GC-MS	TG (16:0/16:0/16:0)	LC-MS
Galacturonic acid	GC-MS	TG (16:0/16:0/18:0)	LC-MS
Glucoheptonic acid	GC-MS	TG (16:0/16:0/18:1)	LC-MS
Glucose	GC-MS	TG (16:0/16:0/18:2)	LC-MS
Glycerol	GC-MS	TG (16:0/16:0/18:3)	LC-MS
Glycolic acid	GC-MS	TG (16:0/18:3/18:2)	LC-MS
Heptadecanoic acid	GC-MS	TG (18:0/18:1/16:0)	LC-MS
Hexanoic acid	GC-MS	TG (18:0/18:2/18:3)	LC-MS
Inositol-3-phosphate	GC-MS	TG (18:0/18:3/18:3)	LC-MS
Isomaltose	GC-MS	TG (18:3/18:0/18:3)	LC-MS
Lactulose	GC-MS	TG (18:2/18:2/16:0)	LC-MS
Levoglucosan	GC-MS	TG (18:2/18:3/15:0)	LC-MS
Linoleic acid	GC-MS	TG (18:2/18:3/18:3)	LC-MS
Linolenic acid	GC-MS	TG (18:3/16:0/17:0)	LC-MS
Malonic acid	GC-MS	TG (18:3/16:1/18:3)	LC-MS
Mannitol	GC-MS	TG (18:3/18:0/16:0)	LC-MS
Mannose 1	GC-MS	TG (18:3/18:2/17:0)	LC-MS
Melezitose	GC-MS	TG (18:3/18:3/16:0)	LC-MS
Methoxytryptamine	GC-MS	TG (18:3/18:3/16:3)	LC-MS
Methyl tyrosinate	GC-MS	TG (18:3/18:3/17:0)	LC-MS
Mimosine	GC-MS	TG (18:3/18:3/17:1)	LC-MS
myo-Inositol	GC-MS	TG (18:3/18:3/17:2)	LC-MS
Myosmine	GC-MS	TG (18:3/18:3/18:3)	LC-MS
Myricetin	GC-MS	TG (18:3/18:3/20:3)	LC-MS
Myristic acid	GC-MS	TG (51:6)	LC-MS
N-acetyl-D-mannosamine	GC-MS	TG (52:2)	LC-MS
Neohesperidin	GC-MS	TG (54:4)	LC-MS

Nicotine	GC-MS	TG (54:7)	LC-MS
